# Development of an anaerobic threshold (HRLT, HRVT) estimation equation using the heart rate threshold (HRT) during the treadmill incremental exercise test

**DOI:** 10.20463/jenb.2017.0016

**Published:** 2017-09-30

**Authors:** Joo-ho Ham, Hun-Young Park, Youn-ho Kim, Sang-kon Bae, Byung-hoon Ko, Sang-seok Nam

**Affiliations:** 1. Department of Sports Medicine, Kyung Hee University, Yongin-si Republic of Korea; 2. Samsung Advanced Institute of Technology, Suwon-si Republic of Korea; 3. Performance Activity and Performance Institute, Konkuk University, Seoul Republic of Korea

**Keywords:** Heart rate threshold, Anaerobic threshold, Lactate threshold, Ventilation threshold, Treadmill, Estimation equation

## Abstract

**[Purpose]:**

The purpose of this study was to develop a regression model to estimate the heart rate at the lactate threshold (HR_LT_) and the heart rate at the ventilatory threshold (HR_VT_) using the heart rate threshold (HRT), and to test the validity of the regression model.

**[Methods]:**

We performed a graded exercise test with a treadmill in 220 normal individuals (men: 112, women: 108) aged 20–59 years. HRT, HR_LT_, and HR_VT_ were measured in all subjects. A regression model was developed to estimate HR_LT_ and HR_VT_ using HRT with 70% of the data (men: 79, women: 76) through randomization (7:3), with the Bernoulli trial. The validity of the regression model developed with the remaining 30% of the data (men: 33, women: 32) was also examined.

**[Results]:**

Based on the regression coefficient, we found that the independent variable HRT was a significant variable in all regression models. The adjusted R^2^ of the developed regression models averaged about 70%, and the standard error of estimation of the validity test results was 11 bpm, which is similar to that of the developed model.

**[Conclusion]:**

These results suggest that HRT is a useful parameter for predicting HR_LT_ and HR_VT_.

## INTRODUCTION

The term “customized exercise” is recently gaining popularity. The application of customized exercise means prescribing the frequency, intensity, type, and time of exercise as appropriate for each individual. Many sports scientists argue that the anaerobic threshold (AT) is the most useful measure of personalized exercise intensity^1, 2, 3^.

AT is the time point at which the rate of energy supply from anaerobic metabolism increases during exercise, and it consists of the lactate threshold (LT) and the ventilatory threshold (VT)^4, 5^. Therefore, to use AT as a parameter of customized exercise intensity, the LT or VT should be determined. However, LT determination requires blood collection and VT measurement requires expensive equipment and manpower, making them difficult to measure outside of the laboratory. Therefore, some sports scientists have investigated ways to easily predict AT.

Conconi et al.^6^ proposed a method of estimating AT using the heart rate threshold (HRT). HRT is the deflection point of the heart rate increase during incremental exercise, and many scholars have suggested that HRT can be as useful as LT and VT in estimating the AT^2, 7, 8^. Nam et al.^2^ estimated the AT using HRT during the incremental bicycle ergometer exercise. They reported an adjusted R^2^ of about 72% and emphasized that the estimation method of AT using HRT is practical. However, few studies have suggested a linear relationship between AT and HRT, as the study by Nam et al.^2^.

Therefore, the purpose of this study was to develop a regression equation that estimates AT using HRT for the treadmill maximal exercise load test.

## METHODS

### Subjects

This study included 220 healthy male and female subjects aged 20–59 years. We confirmed the health of the subjects through the physical activity readiness questionnaire (PAR-Q)^9^ and blood pressure measurement. All subjects were informed about the purpose and process of the study, and voluntarily agreed to participate in the study. All procedures followed were in accordance with the ethical standards of the responsible committee on human experimentation and with the Helsinki Declaration.

Male (n = 112) and female (n = 108) subjects were divided in a ratio of 7 : 3 using the Bernoulli trial. Approximately 70% of the divided data (men: 79, women: 76) were used in the development of the AT estimation formula with HRT, and about 30% of the data (men: 33, women: 32) were used for the validity test. The characteristics of the subjects are presented in Table 1.

**Table 1.** Subject characteristics.

	Sex	n	Age (years)	Height (cm)	Weight (kg)	BMI (kg/m_2_)
Regression model	Male	79	37.5 ± 12.4	173.5 ± 6.5	72.3 ± 10.1	24.0 ± 2.6
	Female	76	37.0 ± 13.0	161.2 ± 5.5	59.0 ± 7.1	22.7 ± 2.6
Validity data	Male	33	35.8 ± 10.8	173.5 ± 7.4	72.5 ± 10.3	24.0 ± 2.5
	Female	32	37.3 ± 11.6	161.5 ± 5.5	60.1 ± 8.2	23.1 ± 3.1

### Experimental design

We instructed all subjects to abstain from drinking and to get adequate sleep from 3 days before the measurement. Subjects who arrived in the laboratory rested for 30 min before starting the test.

A treadmill was used in the maximal exercise test, and we determined the HRT, LT, and VT after the test. We used the heart rate at the determined LT or VT time point as the dependent variable, and HRT as the independent variable.

We used about 70% of the data of all subjects for the development of the regression equation and about 30% of the data of all subjects for the validity test of the developed regression equation.

### Measurements

For all tests, the laboratory temperature was 21 ± 2°C and the humidity was 50 ± 5%.

### Incremental maximal exercise test

For the test, subjects wore the mask of a respiratory gas analyzer (Sensor Medics Vmax229, Milan, Italy) on the face and a heart rate monitor (Polar 610i, Kempele, Finland) on the chest area. We measured the stability parameters of the subjects for 2 min in a sitting position and performed an incremental maximal exercise test using a treadmill (TaeHa TH-6000, Seoul, Korea) until exhaustion.

The protocol of the incremental maximal exercise test was a modification of the Bruce protocol, with the aim to achieve two purposes. First, the protocol should have a small incremental loading between stages so that the responses of the body during exercise can be closely observed. Second, the protocol should be available for use in outdoor fields and indoor tracks. We performed a pilot test >10 times to modify the protocol. These pilot tests included 1–10 subjects aged 20–50 years. The modified Bruce protocol was started at 3.6 km/h, the speed was increased by 1.2 km/h every 2 min, and the slope was 0% at all speeds (Table 2).

**Table 2.** Maximal exercise test protocol (modified Bruce protocol).

Time (min)	Speed (km/h)	Grade (%)
0 – 2	3.6	0
2 – 4	4.8	
4 – 6	6.0	
6 – 8	7.2	
8 – 10	8.4	
10 – 12	9.6	
12 – 14	10.8	
14 – 16	12.0	
16 – 18	13.2	
18 – 20	14.4	
20 – 22	15.6	
22 – 24	16.8	
24 – 26	18.0	
26 – 28	19.2	

We finished the exercise test when three or more of the following occurred: (i) when the subject requested to stop the test; (ii) when the current heart rate exceeded 90% of the predicted maximal heart rate; (iii) when the respiratory exchange rate was >1.15; (iv) when the oxygen intake did not change even when the exercise intensity increased^9^.

Oxygen uptake (VO_2_), carbon dioxide production (VCO_2_), respiratory exchange rate, and ventilation (VE) were measured in real time during exercise. The variables were measured every 1 min and the mean value was obtained. The blood lactate concentration and heart rate were measured as described below.

### Blood lactate concentration

Analysis of blood lactate concentration during exercise was performed every 1 min from the start to the end of the exercise test. For the analysis, blood samples were collected from the subjects’ index finger using a heparinized capillary tube through the “finger-tip” method. The blood samples were analyzed using an automatic blood lactate analyzer (YSI-1500; Yellowspring Instruments, Yellowspring, OH, USA). We determined the LT using the result of blood lactate analysis.

### Heart rate

Measurement of heart rate during exercise was performed every 5 s from the start to the end of the exercise test, using a wireless heart rate monitor. The variables were measured every 1 min and the mean value was obtained. We used these data to determine the maximal heart rate (HRmax) and HRT.

### Determination of LT or VT

The data of blood lactate concentration and respiratory gas during the incremental maximal exercise test were used to determine LT and VT.

LT was determined to be the point at which the blood lactate concentration increased sharply from the resting point^3^.

VT was determined according to certain criteria: (i) the point at which VE/ VCO_2_ does not increase but VE/VO_2_ increases; (ii) the point at which VE and VCO_2_ increase abruptly; (iii) the point at which VE increases relative to VO_2_; and (iv) the point at which VCO_2_ increases relative to VO_2_^3^.

After LT and VT were determined, we defined the heart rate at the time point of LT and VT as HR_LT_ and HR_VT_, respectively.

### Determination of HRT

HRT during the incremental maximal exercise test was determined using the maximal distance (Dmax) method^2, 10^.

The procedure for determining the HRT with the Dmax method follows a certain protocol: (i) Draw a hypothetical straight line connecting both ends of the heart rate trajectory. (ii) Measure the distance from the hypothetical straight line to each heart rate point. (iii) The inflection point is determined as the point having the largest measured distance. (iv) The inflection point is named the HRT. It has been reported that the determination rate of the inflection point with the Dmax method is very high^10^.

However, in this study, HRT was defined as the shortest distance from the virtual straight line. This is because of two reasons. First, according to some studies on HRT^10^, the blood lactate concentration was 3–4 mmoL/l^11, 12^, which corresponds to the onset of blood lactate accumulation. Therefore, Nam et al.^2^ pointed out that early detection of HRT and continuation of exercise were difficult. Second, Hofmann et al.^11^ reported that the HR at lactate turn point 2 (2–4 mmoL/L) was largely different among subjects, whereas the HR at the lactate turn point 1 (0–2 mmoL/L) was not.

**Figure 1.**
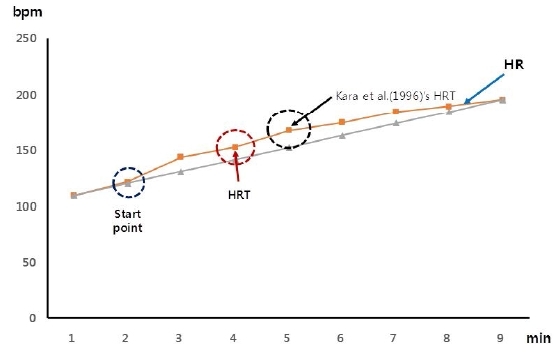
Definition of HRT in this study. HRT = heart rate threshold, HR = heart rate.

The HRT was defined in this study as the first inflection point after setting the point at which the heart rate continuously increases (Figure 1).

### Statistical analysis

The descriptive statistics for each variable were calculated. Different statistical methods were applied according to the purpose of analysis. Statistical analysis was conducted using SPSS 24.0 for Windows and the Excel 2016 program. The level of significance was set at <5%.

### Determination of independent variables

In this study, the detected HRT showed a distinct linearity with the dependent variable (HR_LT_ or HR_VT_). Therefore, we included only HRT as an independent variable considering the simplicity of the regression model.

### Development of the regression model

We used the Bernoulli trial to divide the total subjects in a ratio of about 7:3 (men: 79, women: 76), and developed a regression model for each male and female subject using about 70% of the data. In the development of the regression model, the independent variable was HRT and the dependent variable was HR_LT_ or HR_VT_. In addition, we rigorously conformed to the basic assumptions of the regression model (linearity, independency, continuity, normality, homoscedasticity, autocorrelation, and outlier).

### Validity test of the regression model

The validity of the regression model was tested using approximately 30% of the data (men: 33, women: 32) of the total subjects who had already been divided through the Bernoulli trial and were not included in the development of the regression model. We calculated the predicted value of HRLT or HRVT by substituting the data for the validity test into the regression equation. Finally, we calculated the residuals, the mean error (ME, %), and the standard error of estimation (SEE, bpm), using the difference between the predicted value and the actual measured value of HRLT or HRVT, according to the following formulas^2^:

$$ME\left(\%\right)=\frac{\sum \left(\frac{{HR}_{real}-{HR}_{pred.}}{{HR}_{real}}\times 100\right)}{N}$$

ME: mean error (%)

HRreal: actual measured value of HR_LT_ or HR_VT_

HRpred.: predicted value of H_RLT_ or HR_VT_

Formula 1. The calculation formula for the ME rate for the validity test.

$$SEE\left(bpm\right)=\sqrt{\frac{{\sum \left({HR}_{real}-{HR}_{pred.}\right)}^{2}}{n-2}}$$

SEE: standard error of estimation (bpm)

HRreal: actual measured value of HR_LT_ or HR_VT_

HRpred.: predicted value of HR_LT_ or HR_VT_

Formula 2. The calculation formula of the SEE for the validity test.

## RESULTS

### Deletion of outlier data

The outlier data in the regression model were identified when the absolute value of the standardized residual was 3 or more. In this study, one outlier datum was deleted from the HR_LT_ prediction model of female subjects.

### Maximal exercise capacity and relative ratios of the anaerobic threshold

We analyzed the maximal exercise capacity of the subjects. The maximal oxygen uptake of male subjects was about 50 mL·kg^-1^·min^-1^, the maximal oxygen uptake of female subjects was about 40 mL·kg^-1^·min^-1^, the maximal heart rate of male subjects was 188 bpm, and the maximal heart rate of female subjects was 185 bpm (Table 3).

**Table 3.** Maximal exercise capacity and relative level of anaerobic threshold (AT) for each regression model.

Items	Male model		Female model	
	HR_LT_	HR_VT_	HR_LT_	HR_VT_
VO_2_max (mL·kg^-1^·min^-1^)	49.73 ± 7.04	49.73 ± 7.04	39.75 ± 6.67	39.68 ± 6.68
VO_2_ (mL·kg^-1^·min^-1^)	29.41 ± 8.70	31.08 ± 8.47	23.52 ± 6.59	24.02 ± 7.06
%VO_2_max (%)	58.72 ± 13.13	61.98 ± 11.94	58.82 ± 10.72	60.05 ± 11.33
HRmax (bpm)	188.28 ± 15.04	188.28 ± 15.04	185.11 ± 11.88	184.96 ± 11.89
HR (bpm)	134.77 ± 19.36	138.73 ± 19.97	135.56 ± 21.33	135.84 ± 21.13
%HR (bpm)	71.71 ± 9.28	73.73 ± 8.92	73.06 ± 9.18	73.30 ± 9.37

We also analyzed the relative ratios of AT (LT or VT) to the maximal athletic performance. The oxygen consumption at AT was 59–62% of the maximal oxygen uptake, and the heart rate at AT was 72–74% of the maximal heart rate (Table 3).

### Relative ratios of HRT

We analyzed the relative ratio of HRT to the maximal heart rate. The HRT of male subjects was 68% of the maximal heart rate and the HRT of female subjects was 67% of the maximal heart rate (Table 4).

**Table 4.** Relative level of heart rate threshold (HRT) for each regression model.

Regression model		HRT (bpm)	%HRmax (%)
Male model	HR_LT_	127.2 ± 18.7	67.6 ± 9.0
	HR_VT_	127.2 ± 18.7	67.6 ± 9.0
Female model	HR_LT_	124.2 ± 19.2	67.0 ± 8.7
	HR_VT_	124.4 ± 19.2	67.2 ± 8.7

### Results of the developed regression model

#### Significance of regression models and the independent variable

We tested the significance of the model using the F-test for each regression model developed. As a result, we confirmed that all regression models were statistically significant (Table 5).

**Table 5.** Significance level of the regression coefficient of the independent variable (HRT) for each regression model.

Regression		F-value	Sig.	t-value	Sig.
Male model	HR_LT_	175.431	0.000	13.245	0.000
	HR_VT_	201.299	0.000	14.188	0.000
Female model	HR_LT_	183.351	0.000	13.541	0.000
	HR_VT_	154.513	0.000	12.430	0.000

We used the t-test for each regression model to test the significance of the regression coefficients of the independent variable (HRT). As a result, we found that the regression coefficient of the independent variable was statistically significant in all regression models (Table 5).

### Performance of regression models and regression equations

For each regression model developed, we calculated the adjusted coefficient of determination (adjusted R^2^, adj R^2^) and the SEE. The mean explanatory power of the regression models was about 69.9%, and the mean SEE was about 11.23 bpm (Table 6).

**Table 6.** Adjusted R^2^ (adj R^2^), standard error of estimation (SEE), and regression equation for each regression model.

Regression model		F-value	Sig.	t-value	Sig.
Male model	HR_LT_	0.691	10.76	HRLT = 0.861 × HRT + 25.309	0.000
	HR_VT_	0.720	10.57	HRVT = 0.906 × HRT + 23.544	0.000
Female model	HR_LT_	0.709	11.52	HRLT = 0.936 × HRT + 19.311	0.000
	HR_VT_	0.675	12.05	HRVT = 0.902 × HRT + 23.583	0.000
Mean		0.699	11.23	-	

The final regression equations are presented in (Table 6).

### Validity of regression models

We assessed the validity of the regression model developed using the unused data (30% of the total) in the regression model development. In all regression models, ME was within ±3% for both male and female subjects, and SEE was similar to the regression model developed (about 11 bpm) (Table 7).

**Table 7.** Validity of each regression model

Regression model		HRT (bpm)	%HRmax (%)
Male model	HR_LT_	1.86	11.34
	HR_VT_	0.46	10.47
Female model	HR_LT_	-2.61	10.88
	HR_VT_	-1.64	10.72
Mean		-0.48	10.85

## DISCUSSION

### Goodness of fit of data

In linear regression analysis, it is recommended that outliers are removed because they increase the error of the estimates. Generally, outlier determinations in regression analysis use the absolutely value of standardized residual. In this study, only one outlier was observed in the HR_VT_ prediction model of women. This result implies that the linearity between the independent variable and the dependent variable is clear. Moreover, it is consistent with the previous studys’ claim that there is a relationship between HRT and AT^6, 8^

### Levels of maximal or submaximal exercise capacity

Nam et al.^2^ performed bicycle ergometer tests on subjects similar to those of the present study, and reported that the VO_2_max and HRmax of male subjects were 43 mL·kg^-1^·min^-1^ and 182 bpm, respectively, and those of female subjects were 34 mL·kg^-1^·min^-1^ and 177 bpm, respectively. However, in this study in which a treadmill was used, the VO_2_max and HRmax of male subjects were 50 mL·kg^-1^·min^-1^ and 188 bpm, respectively, and those of female subjects were 40 mL·kg^-1^·min^-1^ and 185 bpm, respectively. The maximal exercise capacity measured in this study was higher than that of Nam et al.'s study^2^, and we considered that this was due to differences in the types of exercise (treadmill vs. bike). In other words, treadmill running involves more wholebody motion than bicycle exercise; thus, the maximal exercise capacity was higher in treadmill running than in bicycle exercise. However, many studies claimed^13, 14^ that maximal exercise capacity is not a good indicator of the fitness level. Therefore, it is necessary to develop a reasonable athletic index.

In this study, VO_2 _at AT was slightly higher than reported by Nam et al.^2^, and heart rate at AT was similar to that of Nam et al.’s study^2^. The results of these comparisons show that VO at AT is high when VO_2_max is high, and heart rate at AT remains the same when HRmax is high. This suggests that AT is a reasonable indicator of exercise intensity^15^ but results may vary depending on the type of exercise. Therefore, AT should be used with caution. If these problems are fundamental because of the type of exercise, we could not explain these problems solely with the results of this study. Therefore, further study is needed on the timing of AT expression according to the type of exercise (or type of test). Kang^16^ measured the AT using an arm ergometer and a bicycle ergometer, respectively. They found a significant difference between the test types in both LT and VT. In addition, Kang^16^ explained that this difference was due to the combination of various factors such as skeletal muscle mobilization, distance between the muscle and heart, isometric kinetic factor, muscle fiber component, motor unit mobilization type, total capillary cross-sectional area, concentration of aerobic enzyme, and mechanical pressure of blood vessels. These explanations suggest that there may be differences in the timing of AT expression according to the type of exercise.

On the other hand, we also considered the possibility that this difference occurred in the measurement process of the AT. However, as the measurement method of LT and VT^3^ is traditionally clear, the possibility of error in the measurement method is not high.

Compared with a previous study^2^, the relative level of HRT was similar but the absolute level was somewhat higher in this study. Further research is needed to determine if the results are due to differences in exercise types, as described above.

### Results of the developed regression models

Significance of the independent variable (HRT)

The regression models of HR_LT_ and HR_VT_ developed in this study were all statistically significant. This is because we strictly adhered to the statistical baseline assumptions of the regression analysis. Moreover, we found that HRT was a statistically significant independent variable in all regression models. These results provide evidence that HRT is a significant predictor of AT (HR_LT_ or HR_VT_).

### Performance of regression models

The average adjusted R^2^ of the regression models developed in this study was about 70%. Considering that the adjusted R^2^ has a lower value than R^2^, it is important that the adjusted R^2^ of this study is about 70%. The reason we chose adjusted R^2^ in this study is to avoid the problem of increasing R^2^ with a larger sample size. The adjusted R^2^ of 70% emphasizes that HRT is useful as a predictor of HR_LT_ and HR_VT_.

The results of this study demonstrate that HRT can be useful in estimating AT during treadmill exercise in male and female subjects aged 20–50 years.

### Results of the validity test

In the present study, the validity of the regression model was analyzed using the data of subjects who were not used in the development of the regression model. The mean error rate was within ±3%. This result is similar to that of Nam et al.’s study^2^ with similar subjects. The SEE of validity test was about 11 bpm, which is similar to the error of the regression model developed in this study (about 11 bpm). Therefore, we confirmed that the regression model developed in this study has statistical significance, good performance, and validity.

## CONCLUSION

The results of this study confirm that HRT is a statistically significant variable in all regression models. Moreover, the adjusted R^2^ of the developed regression models is about 70% on average. In addition, the validity test showed that the SEE was about 11 bpm, which is similar to that of the regression model developed in this study. In summary, HRT is a useful parameter for predicting HR_LT_ and HR_VT_.
